# Web-Based Stress Management Program for University Students in Indonesia: Systematic Cultural Adaptation and Protocol for a Feasibility Study

**DOI:** 10.2196/11493

**Published:** 2019-01-25

**Authors:** Dilfa Juniar, Wouter van Ballegooijen, Eirini Karyotaki, Anneke van Schaik, Jan Passchier, Elena Heber, Dirk Lehr, Sawitri Supardi Sadarjoen, Heleen Riper

**Affiliations:** 1 VU Amsterdam Amsterdam Netherlands; 2 GGZ inGeest Amsterdam Netherlands; 3 GET.ON Institute for Online Health Training Hamburg Germany; 4 Leuphana University Lüneburg Germany; 5 YARSI University Jakarta Indonesia

**Keywords:** internet intervention, stress management, cultural adaptation, feasibility study, low and middle income countries (LMICs), university student, Indonesia

## Abstract

**Background:**

The number of university students experiencing stress is increasing, which often leads to adverse effects such as poor grades, academic probation, and emotional problems. Unfortunately, most of these problems remain untreated because of limited professional resources and fear of stigma. Several Web-based stress management interventions are now available for student populations, but these treatments are not yet available in Indonesia. To make treatment for stress more acceptable in Indonesia, a cultural adaptation process is needed, and part of the process is assessing the feasibility of the adapted intervention.

**Objective:**

This paper describes the first two stages of a cultural adaptation process and the protocol of a feasibility study that will assess the acceptability of a culturally adapted stress management intervention for university students in Indonesia.

**Methods:**

Focus group discussions with Indonesian university students were held, and input from Indonesian psychologists was gathered for developing the adapted intervention. A single-group feasibility study with a pre-post design will be conducted. We will recruit at minimum 50 university students who have an elevated level of stress (Depression, Anxiety, and Stress Scales–42 stress subscale score ≥15), identify themselves as being of Indonesian culture (eg, able to speak Bahasa Indonesia fluently), and are studying at a university in Indonesia. The primary endpoints of this study will be rates of participant satisfaction, system usability, dropout rates, and level of adherence. We will also use qualitative data to assess the adapted intervention more thoroughly. Secondary study endpoints will be quality of life, stress, anxiety, and depression levels. Feasibility parameters (eg, participant satisfaction, system usability, and level of adherence) will be summarized with descriptive statistics. Two-tailed paired within-group t tests will be used to analyze stress, anxiety, depression, and quality of life.

**Results:**

The enrollment of pilot study is currently ongoing. First results are expected to be ready for analysis in the second half of 2019. The project was funded as part of a PhD trajectory in 2015 by the Indonesian Endowment Fund for Education.

**Conclusions:**

This is one of the first studies to assess the feasibility of a culturally adapted Web-based stress management intervention for university students in Indonesia. Strengths and limitations of the study are discussed.

**International Registered Report Identifier (IRRID):**

DERR1-10.2196/11493

## Introduction

Stress is a common phenomenon experienced by many people, including university students. To a certain extent, stress is desirable for human thriving to prevent understimulation [1]. However, ongoing high levels of stress may lead to psychological distress, anxiety, depression, physical illness, substance abuse, and impaired performance at school or work [1,2]. Globally, studies indicate an increasing number of university students experience stress [3-10]. These students are challenged to cope with academic and social demands encountered during their studies and career preparation after university graduation [3,5,11]. Stress experienced by university students can lead to poor grades and academic probation, which in turn may lead to depression, poorer emotional and behavioral skills, social isolation, lower academic performance, and study dropout [12]. Students’ ability to successfully deal with stress during their academic trajectory is an important factor for their academic success and well-being.

A meta-analysis showed that stress management based on cognitive, behavioral, and mindfulness interventions can significantly reduce symptoms of stress and anxiety [13]. Despite existing stress management interventions and the increasing number of university students experiencing general mental health problems [12] including high levels of stress [14], most students do not get help for various reasons [12,14], including limited availability of skilled professionals within universities who can provide counseling for stress management [12,15-17] and fear of the stigma of mental illness if they seek help [12]. Thus, there is a gap between the need for help and the availability of help for university students who are experiencing stress during their studies.

Web-based interventions might encourage university students to seek help for stress-related issues. Compared with traditionally delivered stress-related support, Web-based interventions have the advantages of being more accessible and cost effective and less stigmatizing than traditional face-to-face interventions. Moreover, studies report that most university students are using the internet to seek information and help for emotional and mental health problems [18,19]. Many studies have assessed the clinical effectiveness of Web-based interventions among student populations for a wide range of conditions including stress [20,21], depression and anxiety [21], alcohol misuse [22-24], smoking cessation [25], and obesity [26] with positive results. Furthermore, a meta-analysis showed the potential effectiveness of a Web-based intervention for reducing stress [27]. These studies have mainly been conducted in high-income European countries, the United States, and Australia. By contrast, little is known about the effectiveness of Web-based interventions for stress reduction in low- and middle-income countries such as Indonesia.

A national survey conducted by the Ministry of Health of the Republic of Indonesia estimated the national prevalence rate of psychological distress in Indonesia to be 6% in 2013 [28]. Distress was indicated using the Self-Reporting Questionnaire (SRQ), which consists of 20 questions reflecting symptoms of psychological distress including several items that measure stress (eg, Are you easily tired? Is your daily work suffering? Do you have trouble thinking clearly? [28]). This study included university students but did not report the exact prevalence rates for this population. Although data on national prevalence rates of stress among university students in Indonesia are scarce and related to psychological distress, some local studies among nursing and medical students revealed that most of the students experience stress that has negative consequences for their academic performance and health [29-31]. It is reported that the majority of nursing students were experiencing levels of stress that were moderate (43.4%), mild (30.8%), severe (11.5%), or very severe (1.9%) [30]. Among medical students, a high prevalence was found as well (71%) with students experiencing moderate (54.1%), mild (34.7%), and severe (11.2%) levels of stress [31]. Student willingness to seek counseling inside or outside the university was generally low [32]. Reluctance to disclose problems to a counselor, confidentiality issues, feeling embarrassed if seen going to a counselor, difficulty in finding a counselor or reaching counseling service locations were some of the factors that discouraged Indonesian university students from seeking help [32].

Web-based interventions might increase the help-seeking behavior among Indonesian students due to their easy access and high anonymity level and their 24/7 availability, thereby helping students overcome the perceived stigma of being mentally ill. In Indonesia, this stigma applies to everyone who is seeing a mental health care provider for help even though stress is not a mental illness. Providing Web-based intervention for stress among university students in Indonesia is increasingly feasible because all university students have access to the internet. Internet penetration has improved rapidly in Indonesia and was 54.7% in 2017, with young people the predominant internet users [33]. The field of Web-based interventions is relatively new in Indonesia, and to the best of our knowledge, Web-based stress management for a university student population in Indonesia is not yet available nor are there studies on its effectiveness in terms of stress reduction.

We therefore decided to develop such an intervention for Indonesian university students and evaluate its acceptability and feasibility as a starting point for a future randomized controlled trial (RCT). In order to avoid “reinventing the wheel,” we have chosen an existing evidence-based work-stress–related intervention as a starting point. GET.ON Stress is an evidence-based online stress management intervention for adults appears applicable to a wide range of settings [34,35]. The GET.ON Stress intervention was developed for German employees and is based on the Lazarus transactional model [36]. In this model, coping with stress consists of problem-focused and emotion-focused strategies, which can be considered to be a general framework applicable to many areas of life [37] including the student context [38].

As we wanted to apply this intervention in a context that is different from the target group and culture where the intervention was developed, we decided to culturally adapt it in order to increase the potential acceptability, user satisfaction, and user engagement among our intended target group (ie, Indonesian university students) [39-43]. A Web-based stress management intervention is a novelty in Indonesia and cultural adaptation was therefore recommended [43]. The cultural adaptation process may also increase the probability that the adapted intervention would be more clinically effective than the nonadapted version [44,45]. The evidence addressing this issue is still mixed, however, because in most comparative studies, a culturally adapted intervention is compared with a control condition and not with unchanged versions of the original intervention [43,46].

Details on cultural adaptation methodologies in most studies are not well reported, and this includes the area of Web-based interventions [45]. A meta-analysis on cultural adaptation of Web-based interventions for common mental disorders, including stress, found a wide range in the scope of cultural adaptation across the studies, with language translation and use of metaphors the most frequently recurring applied elements of adaptation [45].

We considered various cultural adaptation methods including the intervention mapping method [39], formative method for adapting psychotherapy [47], and ecological validity model or Bernal Framework [48]. These models and frameworks proposed different methods for carrying out cultural adaptation, although some steps are similar. However, Barrera et al [42] proposed an integrated cultural adaptation method as a consensus derived from the cultural adaptation methods offered in the field. Based on this reasoning, we use as a guideline Barrera’s integrative cultural adaptation model, which consists of five stages: information gathering, preliminary adaptation design, feasibility study, adaptation refinement, and an RCT [42].

In this paper we describe the first two stages, information gathering and preliminary adaptation, and present the protocol of the feasibility study. The remaining stages, including an RCT, will be published in due course.

## Methods

### Stages of the Cultural Adaptation Process

#### Stage 1. Team Setup, Translation, and Information Gathering

A local research team in Indonesia was set up to provide technical support for the research project in Indonesia. The original German version of the GET.ON Stress intervention was translated into English by the intervention’s authors and subsequently translated into Bahasa Indonesia by an independent professional translator who is fluent in both English and Bahasa Indonesia. A process of back translation will be incorporated in a later phase of the project (adaptation refinement, stage 4).

The main aim of stage 1 was to determine which components of the original intervention needed to be modified [42] and explore culturally sensitive aspects related to stress [48].

We conducted 5 focus group discussions (FGDs) aimed at exploring end-user opinions concerning the general impression, look and feel, content, wording, and interface of the GET.ON Stress intervention. We also explored signs of stress the students experienced, idioms related to stress, and how they perceived stress in general. The FGDs comprised 25 Indonesian master’s and PhD students who were studying in the Netherlands, divided into 5 small groups. We recruited these students through announcements made in the Indonesian Students’ Association social media link. The FGDs were held in October 2016 at the VU University in Amsterdam. Each group session lasted for 90 minutes and was led by the principal investigator with help of an assistant. The FGDs yielded the following results: the intervention should be shorter, not contain too much text, use more everyday language that is not too formal, and be more interactive. The signs of stress students mentioned could be categorized into psychological and biological symptoms such as easily irritated, feeling low, headache, and loss of appetite.

A literature search was conducted to find a term that represents stress in Indonesian culture. “ *Banyak pikiran/kepikiran* ” in Bahasa Indonesia (“thinking too much”) was found as an idiom for expressing stress with a negative effect [49]. This was confirmed by the focus group participants. Most participants said that the terms *stres* and *banyak pikiran* are common terms used to define stress among Indonesians. Participants also saw stress as being a less stigmatizing term than depression.

Four bilingual Indonesian psychologists who speak Bahasa Indonesia and English reviewed the first version of the adaptation to identify any potentially problematic features and translation mistakes [42]. Both the English version and the Bahasa Indonesia version of the intervention were reviewed. These psychologists provided information on what they thought was needed to change the intervention in terms of content, structure, and instructions in order for it to be more suitable for Indonesian students. They were also asked whether the therapeutic elements of the original version (ie, problem solving and emotion regulation) would be feasible for the target group [50]. This resulted in several suggestions for change including adaptations of pictures, case examples, metaphors, and examples of activities given in exercises; there was no need expressed for changing the core therapeutic elements. A detailed summary of this stage can be found in Multimedia Appendix 1.

#### Stage 2. Preliminary Adaptation Design

Based on input gathered in stage 1, we modified the original GET.ON Stress intervention, balancing fidelity to the original intervention with the necessary modification of the intervention to the Indonesian cultural context [51-54]. In doing so, we kept the core elements—theory, internal logic processes, and main content—of the GET.ON Stress program [39,51]. However, due to technological, budgetary, and time constraints, the following suggestions for change from the focus group participants and review psychologists could not be effectuated: share function to social media, tangible rewards for participants, more animation, live session with eCoach, offline accessibility of the intervention, speech-to-text feature, use of a mobile app, and option for participants to choose which session they want to start with.

Many adaptations were made. We shortened the intervention by compressing problem-solving modules 2 and 3 in GET.ON Stress into one module in the adapted intervention. We deleted the imaginary traveling element and the souvenir element, designed to motivate participants, after FGD results indicated this might not be suitable for our target population. We made some changes related to pictures, case examples, metaphors, and examples of activities given in exercises to be more suitable to the Indonesian context. We also tried to use more everyday language in each module. Due to the limitations of internet connections in Indonesia, we omitted videos and included slide shows instead. Detailed examples of these changes can be found in Multimedia Appendix 2.

**Figure 1:**
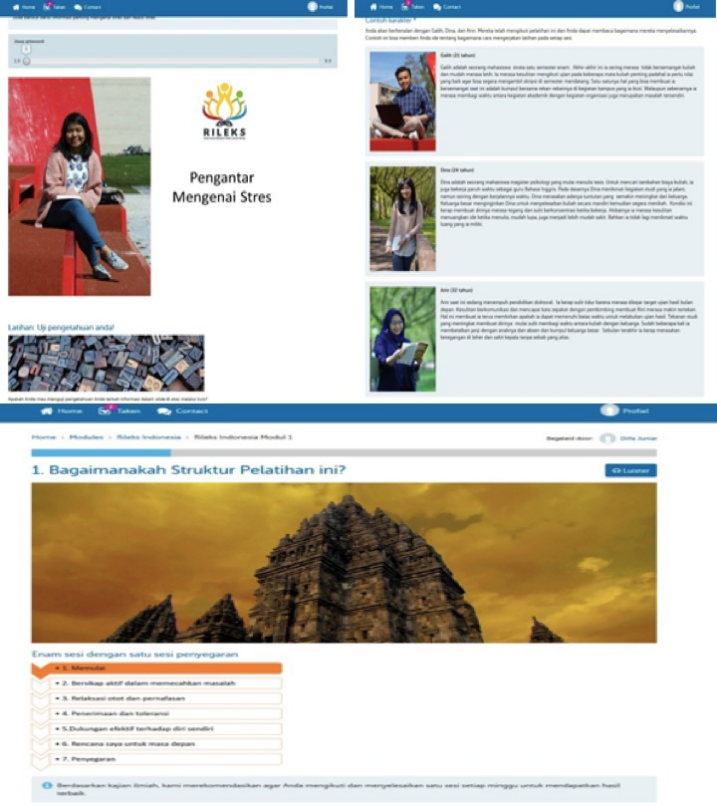
Screenshots from Rileks.

We named the adapted intervention Rileks, which has a meaning similar to *relax* in English and is an abbreviation of “ *intervensi melalui web untuk stres* ” (Web-based intervention for stress). Rileks consists of 6 weekly sessions: psychoeducation, problem solving, emotional regulation (muscle and breathing relaxation, acceptance and tolerance of emotions, and effective self-support), and future planning. The final module is an optional booster session that can be accessed 4 weeks after completing the intervention. Screenshots from Rileks can be found in Figure 1.

eCoach support, asynchronous email communication, is retained in Rileks. During the intervention, each participant will receive feedback on the exercises within 2 days of completing a session. Feedback will be given by eCoaches, Indonesian psychologists who have been trained by the principal investigator. For this training, the standardized GET.ON Stress feedback guide will be used (written communication, E Heber, PhD, 2013). Communication between participant and eCoach will take place on a secure Web-based platform to which both participant and eCoach will have access based on their email addresses and passwords. In addition, participants will receive an email reminder if they have not completed a session within 7 days.

#### Stage 3. Protocol of the Feasibility Study

We will conduct a feasibility study aiming to evaluate the acceptability and feasibility of Rileks among university students in Indonesia using a pretest and posttest design. A secondary aim is to evaluate the success of the study flow (eg, recruitment methods and data collection procedure) in order to set up the RCT in stage 5. If results indicate that Rileks is not yet acceptable and feasible for Indonesian university students, we will complete another information gathering process and adaptation refinement before we proceed with an RCT.

### Study Design

This feasibility study uses a single-group with a pretest (t0) and posttest (t1) design with t1 taking place 10 weeks after t0.

### Sample Size

Due to the aim of our feasibility study, a formal calculation of sample size may not be suitable [55]. Thus, we use convenience sampling in determining sample size. In this study, we intend to include at least 50 participants with a saturation of 75 participants. We believe this number will enable us to obtain sufficiently reliable estimates of our main study parameters.

### Inclusion Criteria

The participants should (1) be aged 19 years or older, (2) be experiencing mild to severe stress, defined as a score of 15 or higher on the 42-item Depression, Anxiety, and Stress Scales (DASS-42), (3) identify themselves as belonging to the Indonesian culture, defined as having the ability to speak Bahasa Indonesia fluently and having grown up with Indonesian customs and lifestyle, and (4) study at a university in Indonesia.

### Procedure

YARSI University, a private university in Jakarta, has agreed to collaborate with this study by allowing us to recruit students for study participation. We will recruit participants by placing standing banners, presenting the project to students, and distributing flyers through student associations in each faculty. In addition to these activities at YARSI, we will also disseminate information via social media such as Facebook and Instagram. Interested students can read detailed information about the study on a dedicated website, where they can also sign up by entering a valid email address. Subsequently, applicants will receive a link to the screening questionnaire. Those who meet the inclusion criteria will be sent a detailed information letter by email about the study and an electronic informed consent form. After the applicants return the electronic informed consent, participants will receive a link to the online baseline questionnaires (t0). All included participants will receive access to the intervention. At the end of each module, we will ask for the participants’ general feedback on the module. Posttreatment assessments (t1) will be scheduled 10 weeks after baseline (t0). In addition, we will invite 10 participants with differing levels of satisfaction for an in-depth interview to evaluate the intervention. It will be clearly indicated to the participants that participation is voluntary and they may discontinue participation at any time and without having to provide any reason for doing so.

### Primary Outcomes

#### Participant Satisfaction

We will use the Client Satisfaction Questionnaire–8 (CSQ-8) [56,57] to assess user perspective on the value of Rileks. The CSQ-8 was translated into Bahasa Indonesia for a previous study in Indonesia [58]. The CSQ-8 is a standardized measure consisting of 8 questions with 4-point response scales (scored 1-4) for a total score range of 8 (great dissatisfaction) to 32 (great satisfaction). Our endpoint of an average score of 20 or higher corresponds to acceptable satisfaction. The CSQ-8 shows high internal consistency with a Cronbach alpha of .93. The CSQ-8 will be administered at posttreatment (t1). In order to obtain more in-depth data related to participant satisfaction, we will invite 10 participants to semistructured interviews after t1.

#### System Usability

We will use the Indonesian version of the System Usability Scale (SUS) [59-61] to assess the usability of the adapted intervention. The SUS comprises 10 questions, and participants will rate the overall usability of all components of the adapted intervention with 5 response options, ranging from strongly disagree to strongly agree. Total scores range from 0 to 100, with higher scores representing higher usability. A score of 70 or higher will be considered adequate as a feasibility criterion [60]. The Indonesian version is considered reliable, with a Cronbach alpha of .84 [61]. The SUS will be administered at posttreatment (t1).

#### Log Data and Adherence to Treatment

Use of the intervention will be measured by tracking the website use automatically: number of sessions completed, time spent per session, and number of log-ins.

Acceptable adherence is defined as 60% [62] or more of participants completing the core online sessions (sessions 1 through 5), where participants will learn the basic principles of problem solving and emotion regulation.

#### eCoach Evaluation

To gain feedback related to eCoach support, we will ask participants questions related to their experience with their eCoaches (eg, how understandable and helpful the feedback from the eCoach was).

#### Rates of Dropout From Study

Study dropout rates are defined as the number of participants who fail to complete the posttreatment assessment.

### Secondary Outcomes

#### Stress, Anxiety, and Depression

To assess the severity of stress, anxiety, and depression, we will use the Indonesian version of the Depression, Anxiety, and Stress Scales (DASS-42) [63]. The DASS-42 is a self-report measure of stress, depression, and anxiety developed by Lovibond and Lovibond [64] consisting of 42 items divided into 3 subscales each containing 14 items. Each item can be answered on a 4-point Likert scale ranging from 0 (did not apply to me at all) to 3 (applied to me very much, or most of the time). The DASS-42 has a total score range from 0 to 42 for each subscale with a higher score indicating a higher degree of severity [65]. The Indonesian version of DASS-42 shows excellent overall reliability with a Cronbach alpha of .95 and high internal consistency in the separate depression, anxiety, and stress subscales (alphas .91, .85, and .88, respectively) [63]. We will administer the Indonesian DASS-42 at t0 and t1.

#### Quality of Life

Quality of life will be measured by the Indonesian version of the World Health Organization abbreviated quality of life assessment (WHOQOL-BREF) [66], which consists of 26 items that measure how the respondent felt in the last 2 weeks, across the 4 domains (ie, physical health, psychological health, social relations, and environment) [66,67]. The WHOQOL-BREF is a valid assessment for use in the Indonesian population as reflected by its internal consistency of .41 to .77 in the 4 domains [68] and its reliability, with intraclass correlation coefficients of .70 to .79 in the 4 domains [66]. The WHOQOL-BREF will be administered at t0 and t1.

#### Demographic Variables

We will request demographic information of each participant, including as age, gender, socioeconomic status, education, marital status, ethnicity, whether they live with parents or live alone, and whether they identify themselves as belonging to the Indonesian culture.

### Analysis

#### Feasibility Parameters

Participant satisfaction (CSQ-8), system usability (SUS), and level of adherence as feasibility parameters will be summarized with descriptive statistics. Point estimates and 95% confidence intervals will be calculated and tested against the feasibility criteria, which were previously defined. Qualitative data, especially related to cultural suitability, will be synthesized and described using thematic analysis, a method for identifying, analyzing, and reporting patterns or themes within data [69].

#### Other Study Parameters

The continuous measures stress, anxiety, depression, and quality of life will be analyzed using 2-tailed paired within-group *t* tests with a level of significance of alpha=.05.

### Ethical Consideration

The study will be conducted in line with the appropriate privacy regulations, and all researchers will follow the Good Clinical Practice guidelines according to Indonesian regulations. The Indonesian ethics committee at YARSI University has given ethical clearance for this research to be conducted (project number: 193/KEP-UY/BIA/VIII/2017).

## Results

The project was funded in 2015 by the Indonesian Endowment Fund for Education as part of the first author’s PhD trajectory. Enrollment for the pilot study is currently ongoing. First results are expected to be submitted by the end of 2019.

## Discussion

To our knowledge, this is the first study to develop and investigate the feasibility of a Web-based stress management intervention for university students in Indonesia. In this paper we described stages 1 and 2 of the cultural adaptation process, as well as the protocol of a feasibility study (ie, stage 3). Data on feasibility will inform further adjustments to the intervention (stage 4) and the potential to conduct an RCT (stage 5), which will provide information on the efficacy of this kind of intervention among Indonesian university students. This study will provide insight into the feasibility of offering a Web-based intervention for stress to university students in Indonesia, which can inform future implementation and dissemination of Web-based interventions in this context.

Implementation and dissemination of evidence-based Web-based mental health care might constitute one of a number of possible strategies to tackle the mental health care gap in low- and middle-income countries where there is disparity between the availability of mental health care providers and the number of individuals in need of mental health care [70-72]. Using internet interventions to deliver self-help and guided psychological interventions is likely to be one possibility for increasing access to mental health care with minimum input from professionals [72]. Rapid increase of internet penetration and the use of technological devices in low- and middle-income countries will accelerate the implementation of a Web-based intervention [73,74]. Furthermore, Web-based mental health care might help to overcome stigma, since patients, including university students, can access mental health care from any location with internet connection [75].

A strength of this study is that we apply a systematic approach to the cultural adaptation process. We involve Indonesian university students as end-user representatives and Indonesian psychologists as lead-user representatives throughout the adaptation stages as we try to integrate “top-down” and “bottom-up” approaches in the adaptation and evaluation process. In doing so, we make sure that Rileks still retains fidelity to the original intervention, while we also take input related to Indonesian cultural context into account [39,42].

The new field and context in which we are working poses challenges for this project. One of those challenges is to include all relevant stakeholders in the developmental process. Ideally, we would have included representatives from the Indonesian professional association in the area of university student mental health as well as policy makers. These stakeholders are considered important for long-term implementation and dissemination in Indonesian mental health care. However, at this stage of the project, involving these stakeholders is not feasible due to time constraints. Another challenge is funding. The platform we use is relatively affordable by Western European standards; however, it is still too expensive if we want to implement Rileks in Indonesia. Thus, with the limited funding that we had, we were restricted in incorporating all the input gathered from stage 1 into stage 2 (preliminary adaptation design).

Despite these limitations, we hope that this study can contribute to the development of student mental health treatment in Indonesia in general. More specifically for university students in Indonesia, we hope this kind of intervention will help to overcome discouraging factors in help-seeking behavior among those who are experiencing stress during their studies and ultimately reduce the stress levels experienced.

## Appendix

Multimedia Appendix 1 Stage 1. Information gathering.

Multimedia Appendix 2 Stage 2. Preliminary adaptation design.

## Abbreviations

CSQ-8: Client Satisfaction Questionniare–8
DASS-42: Depression, Anxiety, and Stress Scales–42
FGD: focus group discussion
RCT: randomized controlled trial
SUS: System Usability Scale
WHOQOL-BREF: World Health Organization abbreviated quality of life assessment
