# Supplementary material for: Web-Based Stress Management Program for University Students in Indonesia: Systematic Cultural Adaptation and Protocol for a Feasibility Study
Source: JMIR Res Protoc. 2019 Jan 25;8(1):e11493. doi: 10.2196/11493 (PMC6367666; doi:10.2196/11493)
Supplement: Multimedia Appendix 1 [file resprot_v8i1e11493_app1.pdf]

Gender (n, %)		
	Male	13 (52)
	Female	12 (48)
Age (n, %)		
	23	3 (12)
	24	3 (12)
	25	5 (20)
	26	4 (16)
	27	3 (12)
	28	2 (8)
	29	1 (4)
	30	2 (4)
	32	3 (4)
	34	4 (4)
	35	5 (4)
Graduated level of education (n, %)		
	Bachelor	15 (60)
	Master	10 (40)

Stage 1. Information gathering

Cluster	Questions	Summary of responses from end-user representatives (Indonesian unversity students)	Summary of responses from lead-user representatives (psychologists)
1. General	To what extend does the intervention suitable for Indonesian university students?	It may suitable for university students because most of them have access to internet. A web-based intervention also makes it possible for the students to access it anywhere and at anytime. However, there are some possibilities that some students are having low interest in this kind of intervention because they would prefer direct social interaction.	It has chanses to be one of psychological help for university students because most of them are internet and technology savy. However, some adaptation might need to be done.
2. Look and feel	How is your impression of the intervention?	General impression are it has a "clean cut", and it looks professional. However, there are many things could be improved, such as interactivity, more audiovisuals, less text, use of infographics, and a possibility of customized look for each individual.	It looks professional, but might need some adjustment in order to make it more interesting. Opinion from end-user representatives will be relevant to use. However, it should be carefully done because too much of "fun things" might make the intervention looks unprofessional.
3. Condition of interest and engagement	What make you want to use this kind of intervention?	Testimonial from previous user, endorsement from well known public figure, reward, social apreciation, social media involvement, statistic about its effectiveness, curiousity, quizes as starter, part as university program, not time consuming	_
	What will makes you engage in the intervention?	If one feel benefit from at least the first 2 sessions, if there is an online community as support, personalized feedback and apreciation in each module, feedback and reminder, feedback from avatar, reminder and personal profile (accomplishment graph), a good eCoach service, guided, involving significant others if possible.	_
	Which barriers can you think of that would keep you from starting with the intervention?	If the intervention is time consuming, busy with other activities, bad internet connection, web load taking too much time.	_
	Which barriers can you think of that would keep you from completing the intervention?	If the stress is gone, if one already feel better, no internet data service, no internet connectivity, no improvement/ effect after 2 or 3 modules, time consuming, busy	_
	How much time per week would you want to spend on this?	One or two times a week, with duration: 15 minutes per module; 30 minutes per module; 10 minutes per module; 40 minutes per module.	Providing an intervention consist of six modules migh more suitable. In many cases, based on professional experience in face to face therapy, only few clients in Indonesia would attend more than six sessions.
	If you were a participant, would you prefer to be guided or unguided? If guided, to what extent and by whom?	Guided by psychologist who provides feedback and gives reminder. If possible, also provide real time eCoach	For case of stress, eCoach might as well be psychologist, or trained lay counselor
4. Content and cultural adaptation	What do you think about the subjects presented in the modules? (i.e. problem solving and relaxation)	The subjects presented in the modules are still suitable for common Indonesian university students.	Problem solving and emotion regulation are still applicable among Indonesian to manage their stress.
	Do you think the intervention is missing something if we want to use it among Indonesian university students? If so, what?	Support group for participants, audio input option so participants can record their voice instead of typing when doing exercises. Some religious aspect might be added as an activity example or even an optional module.	Social support might take into account. However, this should be applied carefully because in reality people with common psychological issues still afraid of stigma from society. Religious aspect might be added in some examples in the modules.
	What aspect do we need to change related to Indonesian culture ? (i.e. metaphors, case examples, pictures, etc)	Language (use Bahasa Indonesia); use picture that represent Indonesia e.g Indonesian nature, Indonesian university students as model, etc; Indonesian psychologist as eCoach; Using case example that represent problems faced by common Indonesian university students.	In general, agree with end-user opinion. Moreover, some detail such as methapor should be adjusted if possible. Providing example of activities that are more relevant to do in Indonesia.
	Do you know any idiom of stress used among Indonesian?	Stress is a term that is common to use among university students in Indonesia. It is considered less stigmatizing to the term "depression". However, some people also express themselves with saying "Banyak pikiran" when they are stress	The term stress is common to use among Indonesian. It is used interchangeably with the term "distress". In general when one said "stress" more likely it means stress which has negative effect (distress). It still consider save to approach participants using the term "stress". However, more explanation on symptoms of stress would be more useful to approach target group.
	What are signs of stress you have had experienced?	The signs of stress students mentioned could be categorized into psychological and biological symptoms such as: easily irritated, feeling low, headache and loss of appetite, etc	_
5. Wording	What do you think about the language or expression used in the intervention ? is it understandable ?	It is still understandable, however it is too formal. It doesn't make people enthusiastic to following the module	In general it is understandable. Making the new module in more nonformal language and simple sentences would be more suitable for university students.
6. Technology	What interface would be more suitable for Indonesian university students?	Preferably application. However, a web-based intervention is still acceptable especially if it is mobile friendly	_
	Which special features would you like to see in the intervention (e.g. video, doodle, case examples etc)	Option to access it offline; Real time feature with eCoach; Avatar greeting in Bahasa Indonesia; Reward system	_
