# Supplementary figures and images for: Web-Based Stress Management Program for University Students in Indonesia: Systematic Cultural Adaptation and Protocol for a Feasibility Study
Source: JMIR Res Protoc. 2019 Jan 25;8(1):e11493. doi: 10.2196/11493 (PMC6367666; doi:10.2196/11493)

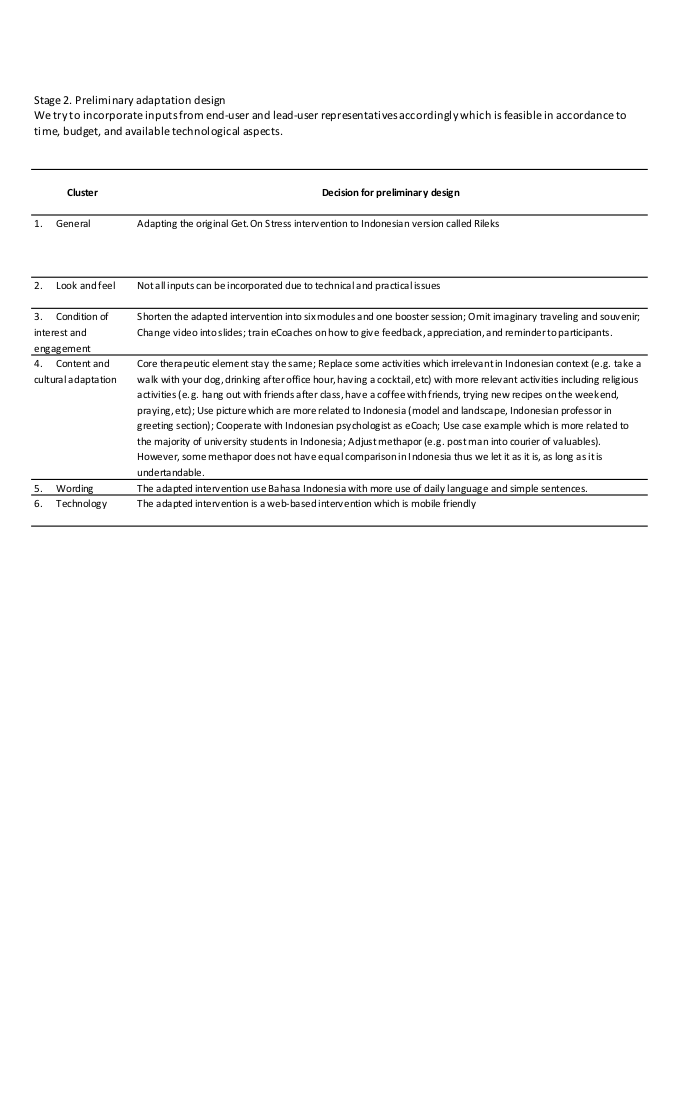

Supplement: Multimedia Appendix 2 [file resprot_v8i1e11493_app2.png]
